# Addressing Asia’s fast growing diabetes epidemic

**DOI:** 10.2471/BLT.17.020817

**Published:** 2017-08-01

## Abstract

Hampered by shortages of resources, specialized services and skilled health workers, India and other countries in south-east Asia are scrambling to respond to type 2 diabetes epidemics. Sophie Cousins reports.

During a visit to his native village outside the Indian city of Chennai, steel plant worker K Shankar (not his real name), 51, dropped in at Dr Mohan’s Diabetes Specialities Centre for a check-up, where he discovered his blood sugar was high, though not quite high enough to be type 2 diabetes.

“They advised me to change my lifestyle to avoid diabetes,” he says. Shankar used to eat a lot of white rice – a large part of the diet of India’s 1.3 billion population – but has since switched to smaller quantities of brown rice with lots of vegetables. He also goes for a walk twice a day.

Set up in 1991, the centre is part of a network of 32 private clinics offering care for some 400 000 diabetes patients across nine Indian states. The centre has also become a World Health Organization (WHO) collaborating centre.

Diabetes is a chronic disease that occurs when either the pancreas doesn’t produce enough insulin (type 1) or when the body can’t effectively use the insulin it produces (type 2).

An estimated 422 million adults were living with diabetes in 2014, according to WHO’s *Global report on diabetes 2016*.

Between 1980 and 2014, the global prevalence of diabetes nearly doubled from 4.7% to 8.5%, with most of the new cases in low- and middle-income countries.

WHO’s South-East Asian Region has seen a recent dramatic increase in diabetes. An estimated 96 million people have diabetes in the region, 90% of whom have type 2, which is preventable. 

However, half of those cases remain undiagnosed, underscoring the need for rapid, low-cost solutions to reach the region’s underserved areas.

Dr Gampo Dorji, technical officer for noncommunicable diseases (NCDs) with WHO’s Regional Office for South-East Asia in New Delhi, notes that the life-style changes associated with rapid urbanization, mechanized transportation, increased consumption of processed foods and physical inactivity are fuelling the epidemic.

He is also concerned that people in the region are developing type 2 diabetes at earlier ages. For Dorji and others who have studied the epidemic, the reasons for the region’s steep increases remain a mystery.

“First the population of south Asia seems to produce less insulin than people in other regions and, secondly, once their glucose levels go up to pre-diabetes levels, they quickly develop diabetes. Why this is the case, we don’t know,” says KM Venkat Narayan, a leading diabetes researcher from the Rollins School of Public Health at Emory University in the United States of America.

“The work is just beginning. We hope that by 2025 good progress will have been made.”

Gampo Dorji

In response to these rapidly growing rates of diabetes and other NCDs including cardiovascular disease and cancer, WHO developed the *Action plan for the prevention and control of NCDs* *in South-East Asia 2013–2020*, which provides a roadmap for implementing policies and programmes at regional and national levels.

The plan calls for a 25% reduction in overall premature mortality from diabetes and other NCDs, and to halt the rise in obesity and diabetes by 2020.

Dorji says most countries in the region have developed their own action plans and are implementing regional plans aimed at expanding diabetes screening at the primary health-care level, training health-care workers and taking prevention measures.

“But the work is just beginning,” he says. “We hope that by 2025 good progress will have been made.”

Two examples are India’s *National programme for prevention and control of cancers, diabetes, cardiovascular diseases and stroke *and Sri Lanka’s *National multisectoral action plan for the prevention and control of NCDs 2016–2020*.

Sri Lanka is addressing the lack of structured screening at the primary health-care level by establishing hundreds of healthy lifestyle centres. Today more than 800 such centres across the country screen for high blood pressure, blood sugar and cholesterol, and overweight and obesity that are risk factors for diabetes and cardiovascular disease.

Depending on patients’ risk, they are either referred to specialized medical clinics for further management or given counselling on healthy living.

For Dr Nalika Gunawardena, who works in the WHO Country Office in Sri Lanka, a major challenge is getting people who feel healthy to get screened.

“People who feel well are not keen to go for a check-up, while some are scared to come, in case they find something and have to start treatment,” she says.

Men are less likely to come for check-ups than women because the centres are mainly open during office hours when most men work. As a remedial measure, the centres are now providing mobile services in workplaces to reach more men.

“There are other gaps in such services,” Dorji says. “We need practical sessions on lifestyle-changing interventions to give people advice on healthy diet and activity, we need a system to follow up people who have come for screening and more trained health-care workers.”

Meanwhile, India’s national programme launched in 2010, focuses on early diagnosis and management of diabetes, human resource development and the promotion of healthy lifestyles.

Narayan says that while awareness around diabetes is high in India, the health-care system is struggling to cope with the disease burden, especially in terms of screening and treatment for complications, particularly in rural areas. 

“The horrible consequences of diabetes cannot be countered without government investment in strong health systems that can deliver prevention and care of high quality to large populations,” Narayan says.

“You can go around testing people, but that’s only the first step. Then you need the capacity and system to deliver good quality care.”

In rural areas, where 70% of India’s population lives, the delivery of diabetes screening, treatment and preventive services is hampered by a lack of skilled health workers, frequent drug stock-outs and long distances to reach health services. Meanwhile, care in urban areas is dominated by the private sector, which is not accessible for everyone in need because of its prohibitive cost.

Given India’s huge rural population and shortages of physicians in such areas, Narayan suggests leveraging technology and making better use of the country’s large network of community health workers known as accredited social health activists (ASHAs) who work as health educators and promoters in their own communities.

Mohan’s staff use telemedicine to support these community health workers in villages across the state of Tamil Nadu, when they screen people for diabetes.

India’s community health workers hold great potential since they can care for diabetes patients in their homes, ensuring that their glucose, blood pressure and lipid levels are under control and can refer them to a physician when needed.

“There’s very strong evidence that if you treat people with diabetes effectively, you can reduce complications and improve quality of life. Effective treatment and follow up by ASHA workers should be implemented more widely,” Dorji says.

Neighbouring Nepal, which has been fraught by political upheaval and natural disasters, is also struggling with a rapidly growing diabetes epidemic.

Deaths from diabetes and other NCDs, including cardiovascular diseases, accounted for 51% of all deaths in 2010 and this figure rose to 60% in 2014, according to national statistics.

However, Dr Buddha Basnyat, director of the Oxford University Clinical Research Unit at Patan Hospital in Nepal, notes that the precise burden of diabetes in the country remains unknown.

In 2014, the Nepali government introduced the *Multisectoral action plan for the prevention and control of NCDs (2014−2020)*.

The goal is to strengthen primary health care by implementing cost-effective interventions for the early detection and management of diabetes.

“It will take a long time to implement policy goals on the ground, especially because there are so many competing problems with inadequate budget allocation,” Basnyat says, citing challenges including the lack of funding and health professionals, and drug stock-outs.

For Narayan, the current drive in many countries to provide treatment and care is vital but should not eclipse efforts to prevent diabetes occurring in the first place. 

That is not easy because prevention measures go beyond the scope of the health sector – reaching into transport, urban planning, economic and fiscal policies – and require strong political commitment. Some countries in the region, such as Sri Lanka and Bhutan, are considering levying a sugar tax, Dorji says.

“Governments need to plan cities to make them walking and cycling friendly so that people get enough exercise naturally,” Narayan says. “They also need to redesign subsidies to make healthy foods more affordable.” 

**Figure:**
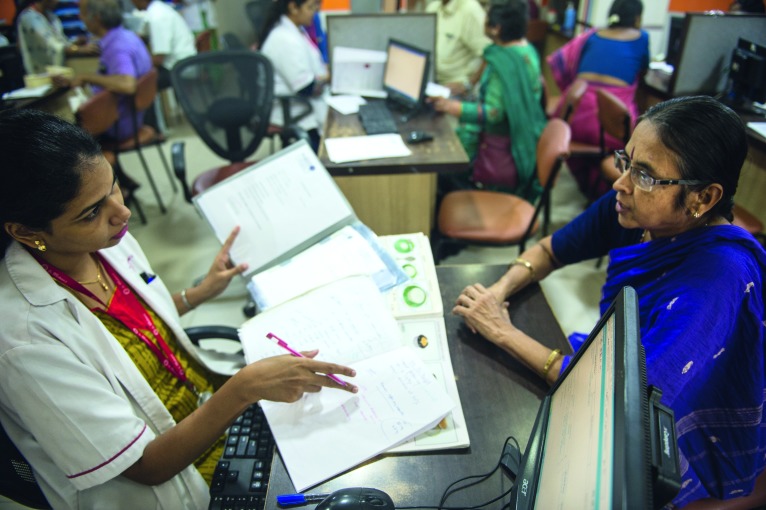
A diabetes nutrition counselling session at Dr Mohan’s Diabetes Specialities Centre in Chennai.

Martin Bader

**Figure:**
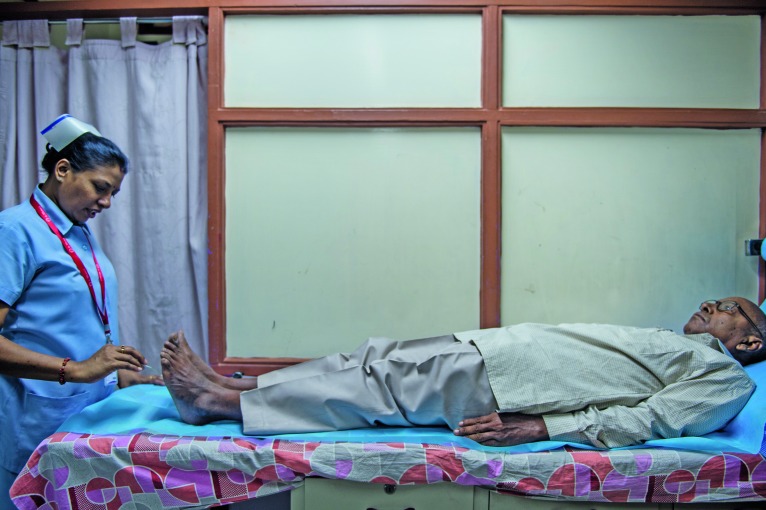
A nurse at Dr Mohan’s Diabetes Specialities Centre checks a patient’s feet for symptoms of nerve damage caused by poorly controlled diabetes.

Martin Bader

